# Effect of individual and provincial levels on stress among general population during the COVID-19 pandemic: a multilevel analysis using mental health survey in Thailand

**DOI:** 10.3389/fpubh.2025.1587819

**Published:** 2025-08-29

**Authors:** Pankaew Tantirattanakulchai, Nuchanad Hounnaklang, Tiwa Mahaprom, Aphichaya Polrak, Nada Lukkahatai

**Affiliations:** ^1^College of Public Health Sciences, Chulalongkorn University, Bangkok, Thailand; ^2^Boromarajonani College of Nursing Chiang Mai, Faculty of Nursing, Praboromarajchanok Institute, Chiang Mai, Thailand; ^3^Department of Mental Health, Prasrimahabhodi Hospital, Ubon Ratchathani, Thailand; ^4^School of Nursing, Johns Hopkins University, Baltimore, MD, United States

**Keywords:** stress, COVID-19, individual-level, provincial-level, multilevel analysis, Thailand

## Abstract

**Background:**

The differential effects of the COVID-19 pandemic across various regions and demographic groups underscore the importance of comprehending the role of social determinants of health (SDOH) in shaping mental health disparities. This research seeks to investigate the influence of both individual-level and provincial-level factors on stress experienced by the Thai general population during the second wave of the pandemic. By incorporating the SDOH conceptual framework within a multi-level modeling approach. The focus is on understanding the impact of these factors on mental health concerns, which have significantly increased due to the pandemic.

**Methods:**

This study is a secondary data analysis of data collected between December 17, 2020, and February 23, 2021, from the Department of Mental Health, Ministry of Public Health, Thailand. Data for the provincial-level including COVID-19 zone and population density, with 258,830 participants nested within the 77 provinces. Stress was measured with 5-item of Srithanya stress test (ST-5). Multilevel analysis was carried out in HLM 8.2 software.

**Results:**

The study found that individual-level factors including male respondents, unemployed, financial hardship, chronic underlying diseases, having older and/or children in family, and having bedridden patients in family tended to report higher stress scores. While, older age respondents, contracted with COVID-19 infection in self and/or family, and having greater resilience showed the lower stress scores. After adjustment for individual-level factors, the respondents who lived in higher and strict control area, and higher population density reported higher stress scores.

**Conclusion:**

Individual and provincial factors appear to influence elevated stress levels. Our research indicates that the government should establish effective resilience promotion programs to mitigate the impact of stress on severe physical and psychological illnesses. Implementing these measures will improve the mental health and well-being of the general population and enhance the country’s preparedness for future pandemics or emerging outbreaks.

## 1 Introduction

COVID-19 was declared a global pandemic in March 2020 (1, 2), with Thailand facing its first wave through superspreading events in nightclubs and boxing stadiums (3). A more severe second wave occurred between mid-December 2020 and late February 2021, driven by outbreaks among migrant workers and illegal border crossings. This wave saw a sevenfold increase in cases compared to the first (4). Beyond its physical impact, the pandemic significantly threatened global mental health. Evidence indicates a substantial increase in stress-related mental health issues among both the general population and vulnerable groups (5–7), leading to various physical and mental health concerns (8).

The social determinants of health (SDOH) encompass the wide range of conditions in which people are born, grow, work, live, and age, influenced by broader systemic factors such as policies, cultural norms, and economic structures that shape daily life (9). These determinants include socioeconomic status, education, physical environment, employment, social support networks, and access to healthcare, all of which interact to influence health outcomes across populations (10). The impact of SDOH is far-reaching, affecting both communicable diseases, such as those that spread from person to person, and non-communicable diseases, including chronic conditions like diabetes and heart disease. These factors do not operate in isolation; rather, they interact with one another in complex ways that can either support or hinder overall health and well-being. In the context of the COVID-19 pandemic, SDOH has played a significant role in determining how different populations are affected, with individuals in crowded living conditions, with limited access to healthcare, or in jobs that do not allow for remote work experiencing heightened stress levels and adverse health outcomes. This underscores the critical importance of considering SDOH in understanding health disparities and the varying impacts of global health crises on stress levels among different population segments.

In Thailand, investigating the role of SDOH is particularly crucial due to the country’s diverse socio-economic landscape, marked by significant inequalities in access to healthcare, economic resources, and living conditions. The uneven impact of the COVID-19 pandemic across diverse regions and demographic groups emphasizes the necessity of a more thorough examination of the ways in which social determinants shape mental health disparities. Given the stark differences between urban and rural areas and the economic challenges faced by vulnerable populations, a focused examination of SDOH can inform targeted public health interventions that address these disparities effectively.

A multilevel approach to understanding the effects of SDOH on stress during the COVID-19 pandemic provides a comprehensive analysis by considering both individual and provincial-level factors. At the individual level, sociodemographic characteristics such as age, gender, employment status, and chronic illness have been linked to increasing stress during the pandemic (11–14). Financial difficulties are particularly significant, leading to reduced access to healthcare, poorer living conditions, and heightened mental health challenges, aligning with the CSDH’s focus on the economic determinants (15). Chronic diseases further exacerbate mental health issues by reflecting both long-term health status and healthcare accessibility (16). Households with vulnerable members, such as children, the older adults, or those with severe illnesses, face additional stress due to income loss, caregiving responsibilities, and healthcare access challenges (17–19). Resilience, defined as the ability to positively adapt to life’s challenges, has proven to be a crucial factor in alleviating the negative impacts of the pandemic on stress levels (20, 21).

At the provincial level, two key factors—population density and COVID-19 zone classification—emerge as significant in the context of the COVID-19 pandemic. Living in crowded spaces or areas with high population density heightens the risk of COVID-19 infection, leading to increased stress levels (22). The COVID-19 zone classification, implemented by the Thai government, reflects the local epidemiological situation and determines the level of public health measures in each area. The Center for COVID-19 Situation Administration (CCSA) raised the level of preventive measures from surveillance areas to maximum and strict control areas based on the increasing number of new cases during the second wave (23). These zone-based measures, including school closures, business suspensions, and movement restrictions, can significantly impact stress levels among the population (4, 23, 24).

**Figure 1:**
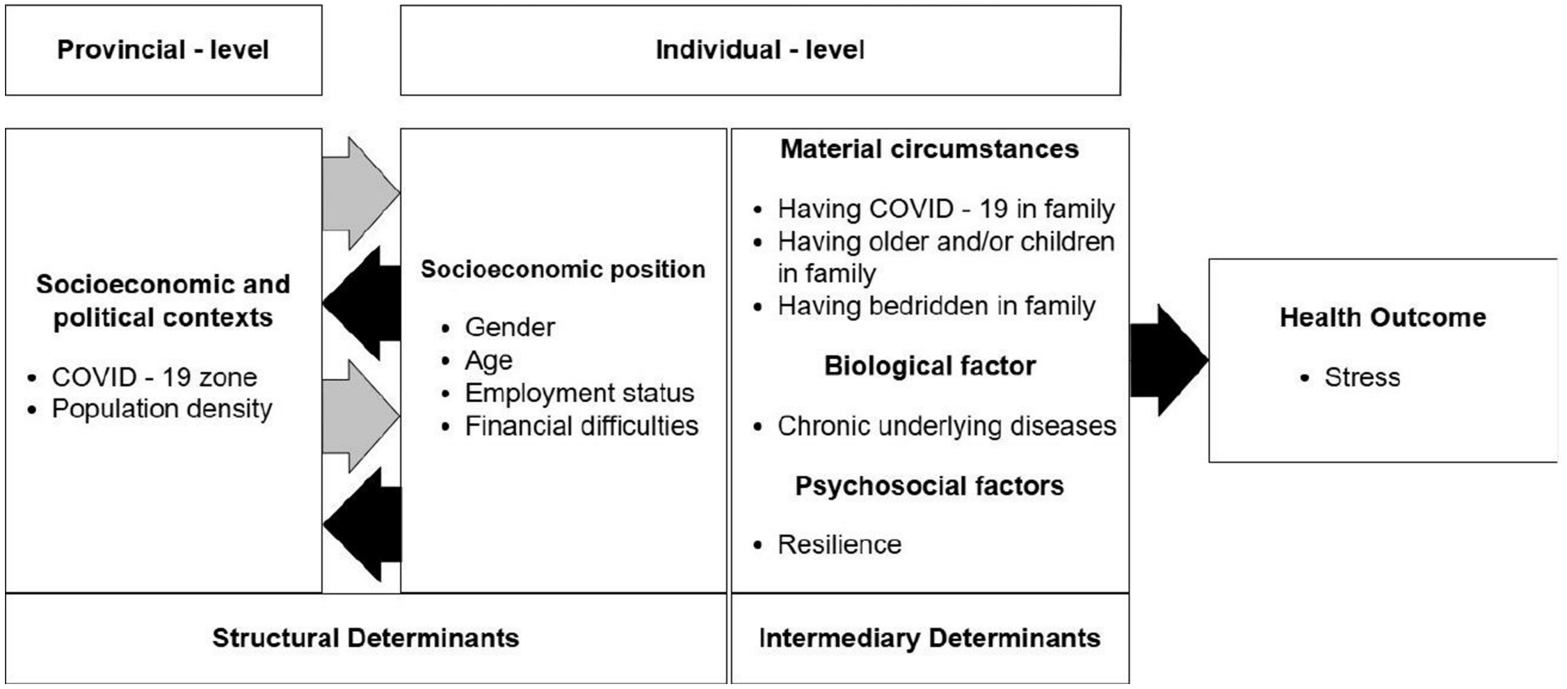
SDOH conceptual framework and integrating it into a multi-level model.

Guided by the World Health Organization (WHO)’s SDOH conceptual framework and integrating it into a multi-level model, our conceptual framework (Figure 1) demonstrates how stress is influenced at two distinct levels—individual and provincial. At the individual level, stress is shaped by a combination of both structural and intermediary determinants. These include socio-economic factors such as age, gender, employment status, and income, which define an individual’s position in society. Additionally, material circumstances like having COVID-19 in the family, caring for older adults or children, and managing bedridden family members are crucial intermediary determinants that directly impact daily life and elevate stress levels. Biological factors, such as chronic underlying diseases, further exacerbate stress by reflecting long-term health vulnerabilities. Psychosocial factors, particularly resilience—the ability to adapt positively to life’s challenges—serve as a critical buffer against stress.

At the provincial level, structural determinants such as COVID-19 zone classification and population density are significant factors that influence stress. These broader socioeconomic and political contexts shape the environment in which individuals live, determining the level of public health measures and the extent of pandemic-related challenges they face. The interaction between these individual and provincial factors ultimately culminates in the overall stress experienced by individuals. By integrating both levels of determinants within this multilevel framework, the model provides a comprehensive understanding of how various factors contribute to stress during the COVID-19 pandemic, emphasizing the complex interplay between individual circumstances and broader structural influences.

This study aims to investigate the influence of both individual-level and provincial-level factors on stress among the Thai general population during the second wave of the COVID-19 pandemic. By integrating the SDOH conceptual framework into a multi-level model approach, the research analyzes the impact of both individual factors, like sociodemographic and chronic illness, and provincial factors, such as population density and COVID-19 zone classification. The objective is to identify key determinants of stress across various regions and groups, offering insights that can inform targeted public health strategies to mitigate mental health disparities during this and future crises.

## 2 Methods

### 2.1 Study population and data source

**Figure 2:**
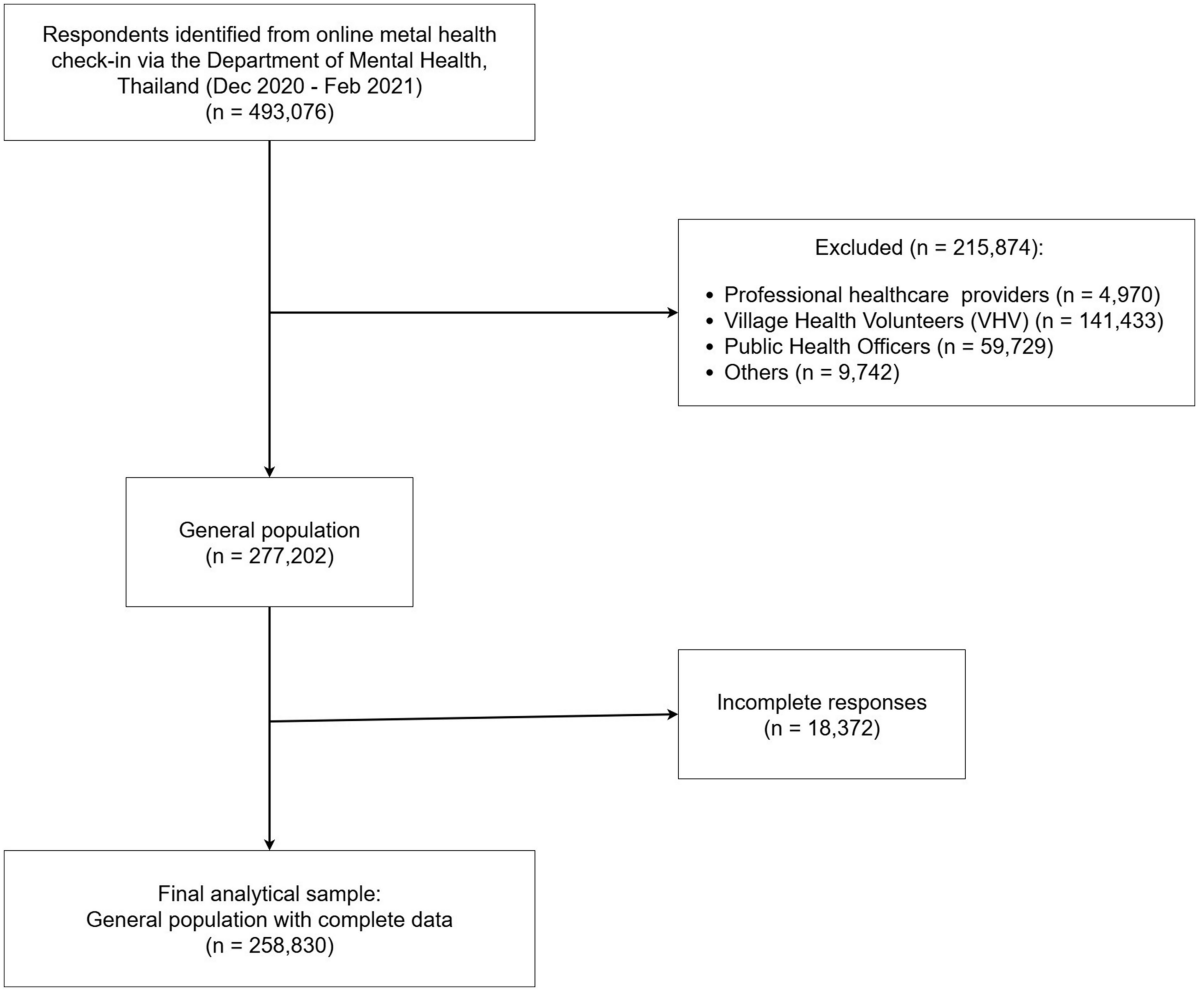
Participants flow diagram.

This study analyzed data collected through an online mental health check-in via the Department of Mental Health, Ministry of Public Health of Thailand website during the second wave of COVID-19 in Thailand between December, 2020 and February, 2021. The dataset initially included 493,076 respondents. After excluding 215,874 individuals who were healthcare professionals or volunteers, and 18,372 who did not provide complete information, the final sample comprised 258,830 individuals from the general population (Figure 2). Provincial-level data on COVID-19 surveillance and control areas were provided by the Center for COVID-19 Situation Administration (CCSA) (23) and population density (people/km^2^) data were obtained from the Official Statistics Registration Systems, Department of Provincial Administration of Thailand (25).

### 2.2 Study variables

#### 2.2.1 Outcome variable

Stress was evaluated using the Srithanya Stress Test (ST-5), an established and broadly recognized tool endorsed by the Department of Mental Health, Ministry of Public Health of Thailand, for measuring stress levels within the Thai population. The ST-5 consists of 5 items that evaluate stress-related symptoms, including sleep problems, loss of concentration, irritability, boredom, and anti-social behavior (26). Each item is evaluated using a 4-point Likert scale: 0 for “Hardly ever or Never” (<1 times/week), 1 for “Sometimes” (1–2 times/week), 2 for “Usually” (3–4 times/week), and 3 for “Always” (5–7 times/week).” The total ST-5 scores span from 0 to 15, with higher scores reflecting a greater frequency or intensity of stress (27).

#### 2.2.2 Individual-level variables

Individual demographic characteristics (e.g., gender, age, employment status, financial hardship, chronic underlying disease, and the presence of vulnerable family members) and resilience were included in the analysis. Resilience was measured using a 3-items scale. Respondents were asked about their ability to overcome obstacles and problems in life, to encourage themselves and feel supported by close relationships, and to manage their own problems and stress over the past 2 weeks. Responses were rated on a 10-point Likert scale, ranging from 1 (less confident) to 10 (very confident). The total resilience score could range from 3 to 30, with higher scores indicating a greater level of resilience (28).

#### 2.2.3 Provincial-level variables

**Figure 3:**
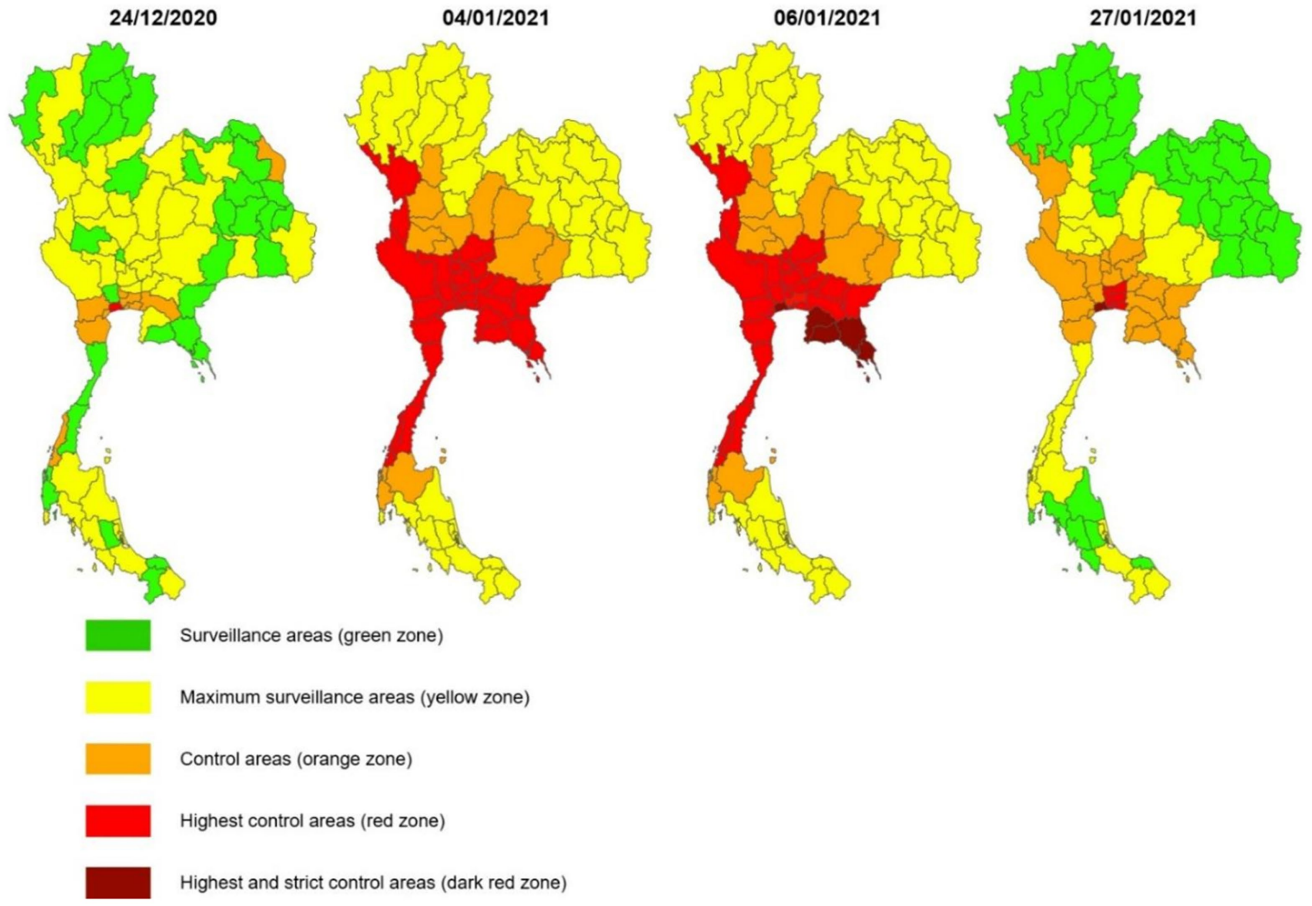
Revolution of provincial zoning and restriction level during the second wave in Thailand.

Population density (measured in people per square kilometer) and COVID-19 risk zones were treated as continuous variables across all 77 provinces in Thailand. The classification of COVID-19 zones followed official guidelines from the Center for COVID-19 Situation Administration (CCSA), which were based on the weekly incidence of newly reported cases per 100,000 population and the scope and intensity of locally implemented public health restriction measures at the local level. Five zone levels were designated: dark red (more than 15 cases or a super-spreader event with over 50 cases), red (5–15 cases or a test positivity rate below 5%), orange (1–5 cases or positivity rate under 2%), yellow (fewer than 1 case and no new cases reported in the preceding 7 days) (Figure 3), and green (no new infections for at least 28 consecutive days) (Figure 3). These classifications guided the implementation of localized restrictions, including curfews, suspension of alcohol sales and domestic travel, and closures of schools, restaurants, markets, entertainment venues, personal care businesses (such as spas, salons, and massage parlors), fitness centers, and other non-essential services (4, 8, 23). Population density data (people/km^2^) were obtained from the Official Statistics Registration Systems, Department of Provincial Administration of Thailand (25).

### 2.3 Statistical analysis

To evaluate model assumptions, a Q–Q plot of the residuals was inspected, revealing an approximately linear trend. This pattern supports the assumption of normality, indicating that the residuals were reasonably normally distributed. Regarding the independence of residuals at both levels, the observed variation in level-1 residuals across level-2 units justified the use of a multilevel modeling approach and suggested that the assumption of residual independence was adequately satisfied.

In this study, we utilized the Srithanya stress scale (ST-5) as the dependent variable, with scores ranging from 0 to 15. We investigated the factors influencing stress scores among the general population during the COVID-19 pandemic using a multilevel approach. The model incorporated two levels: the individual level (first level) nested within provinces (second level). This hierarchical structure allowed us to examine both individual-level and province-level factors contributing to stress scores, accounting for the potential clustering effect within provinces. The analysis was conducted using HLM 8.2 software.

The general form of the multilevel linear regression model is expressed as:

$$Y_{\mathrm{ij}}=\beta_{00}+\beta_{10}X_{\mathrm{ij}}+\beta_{01}Z_{j}+\mu_{0j}+e_{\mathrm{ij}}$$

In this model, *_i_* and *_j_* denote individual and province subscripts, respectively. $X_{\mathrm{ij}}$ represents individual-level variables (e.g., gender, age, employment status), while $Z_{j}$ represents province-level variables (e.g., COVID-19 surveillance and control area, population density). The terms $\mu_{0j}$ and $e_{\mathrm{ij}}$ are residual errors at the province and individual levels, respectively. We assumed zero expectations for $\mu_{0j}$ and $e_{\mathrm{ij}}$ in this model.

The modeling process of this study is as follows: the first step in the analysis is to estimate a null model. The null model (Model 1) is constructed, which only includes a constant term to detect potential contextual variation in stress across provinces. In Model 2, we add individual-level variables. Models 3 and 4 sequentially incorporate province-level variables. Finally, Model 5 includes all individual-level and province-level variables. Statistical significance is set at *p* < 0.05 for all analyses.

## 3 Results

### 3.1 Characteristic of the sample

The study analyzed both individual and provincial-level variables among the 258,830 respondents. Of these 143,309 (55.4%) were male, with a mean age of 48.9 years (SD = 18.5), ranging from 18 to 99 years. The sample included 5.3% who were unemployed, 20.1% facing financial hardship, 14.1% with physical health conditions, 2.0% with mental health conditions, 0.9% from COVID-19 infection in self and/or family, 20.1% from families with older adults and children, and 1.1% with bedridden patients. The average stress score among respondents was 1.7 (from the range of 0 to 15 score), while the average resilience score was 25.8 (from the range of 3 to 30). At the provincial level, the mean COVID-19 zone score was 1.36, and the mean population density was 247.4 people/km^2^ (Table 1).

**Table 1:** Characteristics of the individual and provincial levels variables (*n* = 258,830).

Variables	*n* (%) or mean ± SD
Outcome	
Stress	1.7 ± 2.3
Individual-level	
Gender	
Male	143,309 (55.4)
Female	115,521 (44.6)
Age (years)	48.9 ± 18.5
Unemployed	
No	245,326 (94.7)
Yes	13,594 (5.3)
Financial hardship	
No	206,899 (79.9)
Yes	51,931 (20.1)
Physical health conditions	
No	222,269 (85.9)
Yes	36,561 (14.1)
Mental health conditions	
No	253,668 (98.0)
Yes	5,162 (2.0)
COVID-19 infection in self and/or family	
No	256,471 (99.1)
Yes	2,359 (0.9)
Having older and/or children in family	
No	206,867 (79.9)
Yes	51,963 (20.1)
Having bedridden in family	
No	256,110 (98.9)
Yes	2,720 (1.1)
Resilience	25.8 ± 4.9
Provincial level	
COVID-19 zone	1.36 ± 0.82
Population density (people/km^2^)	247.4 ± 478.2

SD, standard deviation; km^2^, square kilometer.

### 3.2 Multilevel analysis of stress

The multilevel analysis (Table 2) showed that in the initial model (Model 1) where the dependent variable was included. When individual-level variables were added (Model 2), the analysis showed that higher stress scores were significantly associated with being male (*β* = 0.196, *p* < 0.001), unemployed (*β* = 0.728, *p* < 0.001), experiencing financial hardship (*β* = 0.537, p < 0.001), having physical health conditions (*β* = 0.301, *p* < 0.001), having mental health conditions (*β* = 0.391, *p* < 0.001), having older adults and/or children in the family (*β* = 0.199, *p* < 0.001), and having bedridden patients in the family (*β* = 0.792, *p* < 0.001). In contrast, COVID-19 infection in self and/or family (*β* = −0.338, *p* < 0.001), older age (*β* = −0.003, *p* < 0.001) and higher resilience (*β* = −0.181, *p* < 0.001) were associated with lower stress scores. When provincial-level variables were added (Models 3 and 4), the results indicated that living in higher and stricter control areas was associated with higher stress scores (*β* = 0.444, *p* < 0.01). Additionally, higher population density was linked to increased stress scores (*β* = 0.001, *p* < 0.05). In the final model (Model 5), which included all individual and provincial-level variables, most remained significantly associated with stress, except for population density. The intraclass correlation coefficient value of 0.172 in this final model suggests that 17.2% of the variance in stress scores was explained by the combined effects of individual and provincial-level factors.

**Table 2:** Multilevel analysis of individual-level and provincial-level affecting stress.

Variables	Model 1	Model 2	Model 3	Model 4	Model 5
	*β* (SE)				
Individual-level variables					
Gender (ref: female)					
Male		0.196^***^	0.196^***^	0.196^***^	0.196^***^
		(0.008)	(0.008)	(0.008)	(0.008)
Age		−0.003^***^	−0.003^***^	−0.003^***^	−0.003^***^
		(0.000)	(0.000)	(0.000)	(0.000)
Unemployed (ref: no)					
Yes		0.728^***^	0.729^***^	0.728^***^	0.728^***^
		(0.018)	(0.018)	(0.018)	(0.018)
Financial hardship (ref: no)					
Yes		0.537^***^	0.537^***^	0.537^***^	0.537^***^
		(0.010)	(0.010)	(0.010)	(0.010)
Physical health conditions (ref: no)					
Yes		0.301^***^	0.301^***^	0.301^***^	0.301^***^
		(0.012)	(0.012)	(0.012)	(0.012)
Mental health conditions (ref: no)					
Yes		0.391^***^	0.390^***^	0.390^***^	0.390^***^
		(0.029)	(0.029)	(0.029)	(0.029)
COVID-19 infection in self and/or family (ref: no)					
Yes		−0.338^***^	−0.338^***^	−0.337^***^	−0.338^***^
		(0.042)	(0.042)	(0.042)	(0.042)
Having older and/or children in family (ref: no)					
Yes		0.199^***^	0.199^***^	0.199^***^	0.199^***^
		(0.010)	(0.010)	(0.010)	(0.010)
Having bedridden in family (ref: no)					
Yes		0.792^***^	0.792^***^	0.792^***^	0.792^***^
		(0.038)	(0.038)	(0.038)	(0.038)
Resilience		−0.181^***^	−0.181^***^	−0.181^***^	−0.181^***^
		(0.000)	(0.000)	(0.000)	(0.000)
Provincial-level variables					
COVID-19 zone			0.444^**^		0.373^**^
			(0.122)		(0.139)
Population density				0.001^*^	0.001
				(0.000)	(0.000)
Constant	2.985^***^	2.160^***^	2.159^***^	2.159^***^	2.159^***^
	(0.156)	(0.113)	(0.105)	(0.109)	(0.105)
ICC	27.34	19.36	17.29	18.39	17.17
-2LL	−5.747 × 10^5^	−5.484 × 10^5^	−5.483 × 10^5^	−5.484 × 10^5^	−5.484 × 10^5^
*N*	258,830	258,830	258,830	258,830	258,830

^*^p < 0.05, ^**^p < 0.01, ^***^p < 0.001; β, standardized beta; SE standard errors; ICC, Intraclass Correlation Coefficient; −2LL, −2 Log-Likelihood.

Model 1 is the null model.

Model 2 is adjusted for the individual-level variables.

Model 3–4 are adjusted for the province-level variables.

Model 5 is the final model adjusted for all individual-level and province-level variables.

## 4 Discussion

This study was designed to examine how individual-level and provincial-level factors influenced stress among the Thai general population during the second wave of the COVID-19 pandemic by integrating the SDOH conceptual framework into a multi-level model. This study highlights the significant role of the SDOH in individuals’ stress levels during the COVID-19 pandemic, emphasizing on both individual and provincial factors in Thailand. At the individual level, sociodemographic characteristics such as gender, employment status, and financial strain were linked to higher stress, with financial hardship being a particularly important contributor. Physical and mental health conditions, along with caregiving responsibilities, further exacerbated stress, reflecting the compounded burden on vulnerable groups during the pandemic. However, resilience emerged as a crucial protective factor, reducing the impact of stress and underscoring the importance of psychological support during crises. At the provincial level, population density and COVID-19 zone classification played a significant role in influencing stress. Residents in high-density areas and regions with stricter control measures faced increased stress due to the heightened risk of virus transmission and the economic and social restrictions implemented to control the outbreak.

While multiple studies in Canada, Spain, and India reported higher stress in women than men (29–31), we found that Thai men reported higher stress than Thai women, similar to an Israeli study (32). This suggests that traditional gender roles, particularly the expectation for men to be primary financial providers, may have intensified stress during the COVID-19 pandemic. Financial hardship were a key stressor, particularly for individuals experiencing income loss, exacerbated by factors such as low savings and high consumer debt, which led to feelings of powerlessness (33, 34). Thailand’s tourism-dependent economy was especially impacted, with unemployment rates tripling during the pandemic, contributing to a surge in stress levels, especially among young adults (35–37). The link between unemployment and stress underscores the urgent need for accessible mental health services for the unemployed (38, 39). The strong link between financial insecurity, job loss, and stress emphasizes the critical need for accessible mental health services and government-led financial assistance programs to address the mental health impacts of economic instability during crises.

In addition to socioeconomic stressors, caregiving responsibilities and biological vulnerabilities also increased stress levels. Families with older adults or children faced compounded stress due to health concerns and increased caregiving demands, while family caregivers of bedridden individuals experienced significant psychological distress, often due to the loss of external support during the pandemic. Interestingly, contrary to findings from other studies (40–42), individuals in Thailand who were directly affected by COVID-19—either personally or through infected family members—reported lower stress levels compared to those without such exposure. This paradox may be explained by Thailand’s comprehensive pandemic response, which likely played a crucial role in mitigating stress. The government’s multi-faceted strategy included daily updates on COVID-19 cases and regulations through various media channels (43), the implementation of mobile applications for disease tracking (44), and the provision of extensive healthcare coverage through public health insurance programs (45). Additionally, the availability of low-cost COVID-19 insurance policies may have provided financial security, further alleviating stress among the affected population (46). Community-based initiatives were also pivotal in reducing stress. The Village Health Volunteers program, which delivered door-to-door education and support (43), and the Mental Health Crisis Assessment and Treatment Team (MCATT), which offered crucial counseling services, played key roles in offering reliable information and emotional support at the community level (8).

Our study revealed that families with older adults and children experienced higher stress levels during the COVID-19 pandemic, aligning with global trends. The increased vulnerability of older adults to severe COVID-19 outcomes resulted in heightened anxiety and stress for their caregivers (47). Simultaneously, parents faced multiple stressors, including concerns about their children’s health and the challenges of remote learning. The shift to home-based education expanded parental roles, often leading to additional financial pressures. This combination of health concerns, financial strain, and expanded educational responsibilities collectively contributed to elevated stress levels among parents (19, 48). During the pandemic, family caregivers faced additional challenges, such as restricted access to external support and growing concerns about their loved ones’ health (49). During the COVID-19 pandemic, family caregivers faced unique challenges, including restricted access to external support and heightened concerns about their loved ones’ health (50). Pandemic-related restrictions forced caregivers to adjust routines and assume new responsibilities, particularly for high-risk individuals. Research showed that during the early phase of the pandemic, family caregivers reported significantly higher levels of psychological distress and fatigue compared to non-caregivers (51–54). The increased caregiving demands, along with reduced social support, created an environment conducive to elevated stress and mental health issues. Addressing these challenges requires early psychological support for family caregivers, including timely stress assessments, flexible interventions, and establishing trust-based relationships with healthcare providers to help caregivers adapt more effectively, especially during crises like the COVID-19 pandemic.

Individuals with chronic underlying diseases experienced heightened stress, consistent with previous research linking pre-existing conditions to more severe COVID-19 cases and higher mortality rates. In Thailand, the government prioritized vaccination for seven high-risk groups, including those with diabetes, chronic respiratory disease, cardiovascular disease, chronic kidney disease, cancer, obesity, and stroke, to mitigate illness severity among these vulnerable populations. Research consistently shows that COVID-positive patients with chronic conditions, such as psoriasis, multiple sclerosis, and kidney transplant recipients, were more likely to experience higher stress levels compared to those without such conditions (55, 56). Studies from Mexico further highlighted the elevated mortality risk among COVID-19 patients with underlying health conditions, who face a fourfold increased risk of death (57). On the other hand, psychological resilience, the capacity to effectively cope with and recover from challenges, emerged as a crucial factor in mitigating stress during the pandemic (58). Similar to another study (21), we found that high resilient individuals were less likely to experience stress. This aligns with previous research showing that resilience plays a protective role against pandemic-related stress. Resilient individuals exhibited adaptive behaviors, such as recognizing health risks and adhering to preventive measures like mask-wearing (59). A study in Thailand during the early stages of the pandemic also found a link between moderate resilience and lower perceived stress (60), supporting findings from the UK where individuals either maintained resilience or recovered from initial distress within the first 6 months of the pandemic (61). These findings highlight the significant role chronic illnesses play in worsening COVID-19 outcomes and intensifying psychological stress, as individuals with such conditions are acutely aware of their heightened risk. However, the remarkable resilience observed in this study’s population demonstrates an impressive capacity to adapt and recover in the face of adversity, likely helping to mitigate many of the pandemic’s negative psychological effects.

For provincial level factors, population density has been a significant factor in COVID-19 transmission and related stress. Our study found that densely populated areas and stricter COVID control zones were associated with higher stress levels, likely due to increased risk interpersonal interactions and the combination effects of social distancing measures, movement restrictions, and reduced access to public spaces (62). Previous studies, including those from Thailand and South Korea, confirm that densely populated regions saw higher infection rates and more pronounced mental health issues (2, 63). Social isolation, reduced interactions, and limited physical activity during the pandemic further contributed to elevated stress (64, 65). Moreover, residents in densely populated areas may face ongoing challenges in readapting to post-pandemic life, such as returning to work and coping with increased traffic, which could prolong stress even as restrictions ease.

A notable strength of this study is its multilevel approach, which allows for the analysis of both individual- and provincial-level factors influencing stress during the second wave of COVID-19 in Thailand. As one of the first studies to investigate this issue in the Thai context, it provides valuable insights into the interaction between personal characteristics, such as gender, employment status, and chronic illness, and broader structural factors like population density and COVID-19 zone classifications. This approach offers a deeper understanding of how both individual vulnerabilities and environmental conditions contribute to elevated stress levels, informing targeted mental health interventions. Another strength is the use of secondary data from a nationwide mental health survey, which provided a large sample from all provinces, giving the study a comprehensive view of stress across the country. Methodologically, the study employed multilevel analysis to validate findings across different regions, providing robust evidence on factors influencing stress during the pandemic.

However, this study has several limitations. One notable limitation is its reliance on self-reported stress data, which may introduce bias due to subjective interpretation or social desirability effects. Additionally, the cross-sectional study design restricts the ability to establish causal relationships or assess changes in stress levels over time, limiting the capacity to capture the long-term psychological impact of the pandemic. Moreover, the analysis is constrained by the availability of variables in the secondary dataset, potentially omitting other significant factors influencing stress. The online nature of the survey further limits its applicability to short-term assessments, underscoring the need for future longitudinal studies to better understand the prolonged effects of COVID-19-related stress on mental health. Furthermore, the reliance on online data collection poses accessibility concerns, as not all individuals in Thailand have reliable internet access. This digital divide may result in demographic disparities between those who could participate and those who could not, potentially underrepresenting vulnerable populations, including the older adults and individuals with limited access to technology.

The study findings emphasize the need for the government to develop and implement comprehensive policies that mitigate the effects of stress on both physical and psychological well-being, particularly in preparation for future pandemics or emerging public health crises. National mental health education initiatives expanded access to remote healthcare, and integrating stress management techniques into public health programs is essential to enhancing the population’s mental health and resilience. Furthermore, creating support systems for at-risk groups—such as families with vulnerable members, frontline workers, and residents of densely populated areas—should be a priority. Policies should also mandate workplace mental health programs, provide funding for community-based mental health services, and ensure the availability of emergency psychological support during crises. By adopting these measures, governments can more effectively alleviate the adverse impacts of stress, reduce the risk of severe physical and psychological conditions, and enhance the population’s overall well-being. This approach will strengthen preparedness for future public health challenges and improve the nation’s ability to respond to unforeseen outbreaks.

Future research should prioritize establishing a robust and comprehensive national data collection plan, particularly in studying the long-term consequences of post COVID-19 conditions. This plan should include systematic and well-coordinated data gathering efforts that allow for the accurate assessment of prevalence, severity, and varied manifestations across diverse populations. To further enhance the value of research findings, it is also crucial to incorporate additional variables that influence outcomes at higher levels, such as those related to healthcare providers-level and regional-level. By examining these factors before compiling data on a national scale, researchers can gain a more detailed understanding of individuals’ access to health services across various regions, thereby enhancing the development of evidence-based public health strategies and interventions.

## 5 Conclusion

This study demonstrates that during Thailand’s second wave of the COVID-19 pandemic, reported stress levels among the general population were relatively low. However, stress outcomes were significantly shaped by provincial-level characteristics, including COVID-19 risk zone classifications and population density. These results highlight the necessity of integrating regional disparities into public health planning to more effectively safeguard both physical and mental well-being in future health crises. Furthermore, the findings contribute to progress toward the Sustainable Development Goals (SDGs), specifically SDG 3 (Good Health and Well-being) by reinforcing the role of mental health in holistic health promotion, and SDG 10 (Reduced Inequalities) by drawing attention to the importance of addressing geographic and social inequalities in health-related outcomes.

## Data Availability

The datasets presented in this article are not publicly available because they were obtained from the Department of Mental Health, Ministry of Public Health, Thailand, and restrictions apply to their availability. Requests to access the datasets should be directed to the corresponding author, with permission from the Department of Mental Health, Ministry of Public Health, Thailand.
